# The LiaFSR-SpxA2 regulatory axis governs bile salt resistance and virulence in Group B *Streptococcus*

**DOI:** 10.1080/21505594.2026.2721814

**Published:** 2026-08-19

**Authors:** Linhong Wang, Ruoyu Li, Jianhao Lin, Fengyang Li, Yujiao Wu, Yong-An Zhang, Hui Zeng

**Affiliations:** a Hubei Hongshan Laboratory, Hainan Institute, National Key Laboratory of Agricultural Microbiology, College of Fisheries, Huazhong Agricultural University, Wuhan, China; b Hubei Jiangxia Laboratory, Wuhan, China

**Keywords:** GBS, SpxA2, LiaFSR two-component system, bile salt resistance, virulence, *Streptococcus agalactiae*

## Abstract

*Streptococcus agalactiae*, also known as Group B *Streptococcus* (GBS), colonizes the intestinal tract, where it must overcome bile salt-mediated membrane disruption to establish infection. However, the specific molecular mechanisms underlying this resistance remain unclear. In this study, a *Himar*1 transposon screen identified a bile salt hypersensitive *spxA2* (SAHN016_RS09865) mutant. To elucidate how SpxA2 mediates bile salt stress adaptation and promotes GBS pathogenesis, we constructed the *spxA2* deletion mutant Δ*spxA2* by homologous recombination. We found that deletion of *spxA2* significantly compromises membrane stability, as evidenced by a markedly increased negative surface charge and decreased hydrophobicity in the Δ*spxA2* mutant. Under bile salt stress, the Δ*spxA2* mutant exhibited severe membrane depolarization and compromised membrane integrity. Genetic and transcriptional analyses further revealed that the LiaFSR two-component system as the upstream regulator that significantly upregulated *spxA2* transcription in response to bile salt stress, defining a novel LiaFSR-SpxA2 regulatory pathway playing a role in bile salt resistance in GBS. Importantly, this LiaFSR-SpxA2 regulatory axis plays a role in colonization and virulence in a tilapia infection model. Collectively, our work defines the LiaFSR-SpxA2 axis as a central regulator of membrane homeostasis that is critical for GBS to overcome innate host defenses, thereby facilitating intestinal colonization and the progression to systemic disease.

## Introduction

*Streptococcus agalactiae*, also known as Group B *Streptococcus* (GBS) is a globally significant pathogen that poses a threat to both public health and food security through its ability to infect different host species, such as humans, cows, and fish [1–3]. In addition to its role as an opportunistic pathogen, GBS can asymptomatically colonize healthy adults, particularly in the gastrointestinal and genitourinary tracts [4], while newborns are particularly susceptible to invasive GBS disease [5]. Thus, adaptation to the gastrointestinal environment, including tolerance to bile salts, may be important not only for GBS pathogenesis but also for intestinal persistence and colonization [6]. The pathogen establishes and propagates infection by colonizing and invading multiple tissues and organs, breaching the blood–brain barrier, and infiltrating neural systems, which culminates in meningitis and bacterial septicemia [7]. In humans, gastrointestinal colonization of GBS is recognized as a prerequisite for pathogenesis, serving as a gateway for GBS translocation across the intestinal barriers and subsequent systemic invasion [8–10]. Similarly, in tilapia models, GBS also needs to invade the intestinal epithelium to establish systemic infection [11,12].

During intestinal colonization, GBS encounters an adverse environment rich in antibacterial agents, particularly bile salts. Bile salts play an essential role in the intestine by increasing lipolysis, enhancing the intake of fat-soluble vitamins [13], and maintaining intestinal barrier function [14]. Their amphiphilic properties also induce the bacterial cell membrane instability and trigger bacterial death [15]. Bacterial adaptive mechanisms involved in the bile salt resistance can be categorized into the active transport of bile salts from the bacterial cytoplasm via efflux pumps [16], changes in the physicochemical characteristics of the cell wall and membranes [17] and the activity of bile salt hydrolases [18,19]. The deployment of these resistance mechanisms is often tightly controlled by bacterial signal transduction systems, such as two-component systems (TCSs). In GBS, more than 20 TCSs have been identified [20], including *CovR/S* [21] *SaeR/S* [22], *CiaR/H* [23], and *RgfA/C* [24], contribute to host adaptation and virulence regulation by modulating the expression of genes involved in metabolism and pathogenesis. Among these, the LiaFSR TCS is an important regulatory pathway that orchestrates the response to cell membrane stress across Firmicutes, primarily through its downstream effector Spx, a global transcriptional regulator originally identified as a suppressor of *clpP* and *clpX* mutations [25]. The *clpP* and *clpX* genes encode components of the ATP-dependent ClpXP proteolytic system, which is involved in protein quality control and stress adaptation [26]. In most streptococcal species, including GBS, the Spx family comprises two paralogous proteins, SpxA1 and SpxA2, which have evolved distinct regulatory functions. In *Streptococcus mutans*, SpxA1 positively modulated the expression of genes linked to oxidative stress [27], whereas SpxA2 has been shown to influence membrane fatty acid composition and expression of genes involved in cell envelope biogenesis, thus contributing to the maintenance of cell envelope homeostasis under stress conditions [28]. Consistent with this functional divergence, the LiaFSR system has been shown to specifically regulate *spxA*2 expression in response to cell envelope stress induced by peptidoglycan‑targeting antibiotics and cationic antimicrobial peptides (CAMPs) in *S. mutans* [29,30], *S. pyogenes* [31,32], and GBS [33].

While these studies have established the LiaFSR-SpxA2 axis as a key mediator of antibiotic and CAMP resistance, its role in counteracting host‑derived stressors encountered during intestinal colonization remains poorly defined. Notably, bile salts represent a distinct and constitutive intestinal stressor that differs significantly from induced CAMPs in terms of origin, concentration, and physiological context [34]. As amphipathic molecules, bile salts constitutively challenge bacterial membrane integrity in the gut, yet the molecular pathway enabling GBS to resist bile salt‑mediated membrane damage is incompletely understood. A recent study indicates that GBS utilizes cell wall adaptation and transporter upregulation to counteract bile toxicity [35]. However, whether the conserved LiaFSR-SpxA2 regulatory axis functionally contributes to bile salt adaptation and intestinal colonization in GBS has not been experimentally validated.

In this study, screening of a Himar1 transposon mutant library identified SAHN016_RS09865 as a key factor for bile salt resistance in GBS. Sequence alignment revealed that SAHN016_RS09865 is the homolog of SpxA2 in streptococci. Through a combination of genetic and biochemical approaches, we show that SpxA2 maintains membrane homeostasis under bile salt stress and that its expression is directly activated by the LiaFSR system. Furthermore, we establish that the LiaFSR-SpxA2 regulation axis plays a key role in GBS survival in the intestinal environment and virulence in a tilapia infection model. Collectively, our findings reveal a novel physiological role for the LiaFSR-SpxA2 axis in enabling GBS to resist intestinal bile stress, thereby facilitating gut colonization and promoting systemic infection.

## Materials and methods

### Bacterial strains and growth conditions

All bacterial strains and plasmids used in this study are listed in Table S1. The wild-type (WT) GBS strain HN016 was obtained from a clinical case of tilapia meningoencephalitis [36]. GBS WT and mutant strains, along with *Escherichia coli* DH5α and BL21 (DE3), were cultured with shaking 200 rpm at 37°C in Todd Hewitt Broth (THB; Hopebiol, HB0311-3) medium or THB agar plates, and Luria-Bertani (LB; Hopebiol, HB0128) medium or LB agar plates, respectively. Liquid cultures were incubated at 37°C in an intelligent incubator (ZHICHENG, ZWYC-2932) at 200 rpm, while solid cultures were incubated at 37°C for 16 h in a biochemical incubator (TAISITE, SPX-70BIII). Antibiotic supplementation was performed as needed, with both spectinomycin and kanamycin at final concentrations of 100 μg/mL. All optical density (OD_600_) and absorbance measurements were performed using a microplate reader (BioTek Synergy H1).

### Construction of mutant and complemented strains

All primers are listed in Table S2. A Himar1 transposon mutant library was generated as previously described [37]. To screen for mutants with altered bile salt tolerance, the library was replica plated onto THB agar plates with or without 0.015% (w/v) bile salts (Millipore, B8756). Colonies that exhibited normal growth on THB agar but impaired growth on THB agar plates containing bile salts were selected as bile salt sensitive candidates. Inverse PCR was used to identify transposon insertion sites, which were then confirmed by sequencing. The disrupted genes and corresponding insertion sites of the bile-sensitive mutants are listed in Table S3.

The *spxA2* (SAHN016_RS09865) and *liaR* (SAHN016_RS01945) deletion mutants were generated using the pSET4s allelic exchange vector as detailed in our previous work [38]. The successful construction of the Δ*spxA2* and Δ*liaR* mutants was validated through PCR and verified by DNA sequencing (Figure S1a and S1b). For construction of the Δ*spxA2* mutant, the genomic region spanning nucleotides 1,972,725–1,973,141 of the *spxA2* coding sequence (CDS) were deleted. For construction of the Δ*liaR* mutant, the genomic region spanning nucleotides 344,752–345,410 of the *liaR* coding sequence were deleted. For complementation, the CDS of *spxA2* and *liaR* was amplified using primers *spxA2*-complementary-F/R and *liaR*-complementary-F/R respectively, and inserted into the shuttle vector pSET2. In each construct, *spxA2* and *liaR* were expressed constitutively from the *lac* promoter in the pSET2, without the need for induction. The resulting plasmids pSET2-*spxA2* and pSET2-*liaR* were electroporated into the Δ*spxA2* and Δ*liaR* mutants to generate the complemented strains CΔ*spxA2* and CΔ*liaR*, respectively.

### Spot dilution assays

Mid-log phase bacterial cultures (OD_600_ = 0.65) were harvested by centrifugation (5,000 × *g* for 5 min), adjusted to an OD_600_ = 0.65 in phosphate-buffered saline (PBS, pH 7.0), and then subjected to a tenfold serial dilution, yielding a final dilution of 10^6^. Then, 7 μL of each dilution was plated onto THB agar plates supplemented with 0.01% (w/v) or without bile salts followed by incubation at 37 °C for 16 h.

### *In vitro* survival assays

Mid-log phase bacterial cultures (OD_600_ = 0.65) were harvested by centrifugation (5,000 × *g* for 5 min), adjusted to an OD_600_ = 0.65 in PBS (pH 7.0), and exposed to 5 mM H_2_O_2_, 0.25% or 1% bile salts for 30 min. The treated mixtures were then tenfold serially diluted and plated on THB agar plates that were incubated at 37°C for 16 h. Colony-forming units per milliliter (CFU/mL) were counted, and the survival rate was expressed as the CFU count after treatment relative to the CFU count before treatment.

### Bacterial surface charge analysis

The assay for bacterial surface charge was performed as reported previously [39]. Briefly, mid-log phase bacterial cultures (OD_600_ = 0.65) were harvested by centrifugation (5,000 × *g* for 5 min) and adjusted to an OD_600_ = 0.8 in 20 mM morpholinopropane sulfonic acid (MOPS, pH 7.0), followed by the addition of cytochrome *c* (MCE, HY-125857) to 0.2 mg/mL. A cytochrome *c* solution in MOPS buffer, without bacterial cells, was used as negative control and recorded as OD_530 initial_. After incubation at 37°C for 15 min, bacterial cells were harvested by centrifugation (5,000 × *g* for 5 min). The concentration of cytochrome *c* present in the supernatant was measured at 530 nm. The proportion of bound cytochrome *c* was computed as follows: (1-OD_530 supernatant_/OD_530 initial_) ×100%.

### Bacterial surface hydrophobicity analysis

Bacterial surface hydrophobicity was assessed as reported previously [40]. Briefly, mid-log phase GBS cells (OD_600_ = 0.65) were harvested by centrifugation (5,000 × *g* for 5 min), and adjusted to an OD_600_ = 1.0 in phosphate urea magnesium sulfate (PUM) buffer. After mixing 3 mL of the cell suspension with 300 µL of N-hexadecane, the mixture was vortexed vigorously for 30 seconds and then incubated at 30°C for 30 min to obtain complete phase separation. After incubation, the OD_600_ of the lower aqueous phase was carefully measured. The proportion of cell surface hydrophobicity was measured as follows: (1− OD_600 aqueous_/OD_600 initial_) ×100%.

### Intracellular survival assay

The intracellular survival assay of GBS in macrophage was performed as reported previously [41]. Briefly, Dulbecco’s Modified Eagle Medium (DMEM) mixed with 15% fetal bovine serum was used to cultivate RAW264.7 cells (Sunncell, China) into 24-well plates with 2 × 10^5^ cells per well. GBS cells were harvested by centrifugation (5,000 × *g* for 5 min) and adjusted to 4 × 10^6^ CFU/mL in DMEM. Bacteria were then added to the macrophages at a multiplicity of infection (MOI) of 10:1 and incubated for 1 h at 37 °C in a 5% CO_2_ atmosphere to allow phagocytosis. Following infection, cells were washed with PBS and incubated in 500 μL of DMEM containing gentamicin (100 μg/mL) and penicillin G (5 μg/mL) for an extra 1 h at 37 °C in a 5% CO_2_ atmosphere to eliminate extracellular bacteria. The efficacy of the antibiotic treatment was confirmed by plating the supernatant onto THB agar plates, which yielded no bacterial colonies. Following the antibiotic treatment, cells were washed with PBS and incubated with fresh medium for 0 h, 2 h, and 4 h. At each time point, the RAW264.7 macrophage cells were lysed by hypotonic shock using ddH_2_O, and bacterial colonies were enumerated onto THB agar plates. The intracellular survival rate was calculated as follows: (CFU count at time points/CFU count at 0 h) ×100%. Uninfected macrophages processed in parallel were used as a negative control.

### Bacterial membrane potential analysis

Membrane potential of GBS was assessed as reported previously [42], using the cationic dye 3,3′-diethyloxacarbocyanine iodide (DiOC_2_(3); Molecular Probes, D14730). This dye emits green fluorescence (488 → 530 nm) in bacterial cells, and an increase in membrane polarization induces a spectral shift toward red fluorescence (488 → 600 nm). Mid-log phase GBS cells (OD_600_ = 0.65) were harvested and adjusted to an OD_600_ = 0.3 in PBS (pH 7.0). The bacterial suspension was subsequently exposed to 0.25% (w/v) bile salts (experimental group) or PBS (untreated control) for 30 min at room temperature. Following treatment, bacteria were incubated with 30 µM DiOC_2_(3) for 30 min. Membrane potential was evaluated by flow cytometry (BD LSRFortessa) using 488 nm excitation. The fluorescence signals were collected in both FITC and PE channels. The median relative fluorescence intensity (RFI) of red to green fluorescence was calculated and used to compare membrane potential between groups.

### Bacterial membrane permeability assay

Membrane permeability of GBS was determined by propidium iodide (PI; Sigma, P4170) uptake according to established guidelines [43]. PI produces red fluorescence upon binding to nucleic acids by selectively entering cells with compromised membranes [44]. Mid-log phase GBS cells (OD_600_ = 0.65) were harvested and adjusted to an OD_600_ = 0.3 in PBS (pH 7.0). The bacterial suspension was subsequently exposed to 0.25% (w/v) bile salts and 20 μg/mL PI for 30 min at room temperature. The mixture was analyzed by flow cytometry (BD LSRFortessa) using 535 nm excitation. The fluorescence from PI was collected in the PE channel. The extent of membrane damage was expressed as the median fluorescence intensity (MFI) of the PE channel.

### Gene expression analysis

Total RNA was extracted from GBS cultures at OD_600_ = 0.65 using the HiPure Bacterial Mini Kit (Magen, R4181-02). cDNA was synthesized using the HiScript III All-in-one RT SuperMix (Vazyme, R333-01). The primers used for qRT-PCR are listed in Table S2. Quantitative real-time PCR (qRT-PCR) was performed using the SYBR Green PCR Master Mix (Vazyme, Q321-02) on a CFX Connect Real-Time PCR System (BioRad). The expression of *spxA2* gene was normalized to the housekeeping gene *gyrA*, and the 2^−ΔΔCt^ method was used to calculate relative gene expression levels [45].

### Expression and purification of LiaR

The *liaR* gene was amplified by PCR from the genomic DNA of GBS strain HN016 with primers (pet28a-*liaR*-F/R) and cloned into the EcoRI/BamHI sites of pET28a, generating the protein expression plasmid pET28a-*liaR* for producing His-tagged LiaR. For LiaR protein expression, *E. coli* BL21 (DE3) cells transformed with the pET28a-*liaR* were grown in 2 L LB medium supplemented with 100 µg/mL kanamycin at 37°C and shaking at 180 rpm. Protein expression was induced at OD_600_ = 0.6 by adding IPTG to final concentration of 0.5 mM, and the cell culture was further incubated at 37°C for 4 h. Cells were harvested by centrifugation at 6,000 × *g* for 15 min at 4°C and the pellet was resuspended in 100 mL of lysis buffer (20 mM Tris-HCl; 150 mM NaCl; 10% glycerol; pH 7.0) containing a protease inhibitor cocktail (Roche, 11,836,153,001). Cells were lysed by passing the cells twice through a French press at 1,000 psi. The resulting lysates were clarified by high-speed refrigerated centrifugation (Eppendorf, Himac CR21N) at 12,000 × *g* for 70 min at 4°C, and the supernatants were collected. The collected supernatants containing His-tagged LiaR were incubated with Ni-NTA magnetic beads (Yeasen, 20561ES25) for 1 h at room temperature to allow binding of His-tagged protein. After incubation, the supernatants were washed with wash buffer (20 mM Tris-HCl; 150 mM NaCl; 50 mM imidazole; 10% glycerol; pH 7.0). The His-tagged LiaR protein was eluted with elution buffer (20 mM Tris-HCl; 150 mM NaCl; 400 mM imidazole; 10% glycerol; pH 7.0). The purity and identity of the recombinant LiaR protein were analyzed by SDS-PAGE (Figure S2a) and Western-blotting with the His tag antibody (Figure S2b).

### Electrophoretic mobility shift assay (EMSA)

EMSA was used to assess the interaction between the *spxA2* promoter DNA and LiaR [46]. Prior to EMSA, the *spxA2* promoter sequence was analyzed using bacterial promoter prediction program BPROM (http://www.softberry.com/berry.phtml). To identify potential LiaR binding sites, the upstream region of *spxA2* was aligned with the reported LiaR consensus motif using MEME tool (https://meme-suite.org/meme). For EMSA, the promoter regions of the *spxA2* gene were obtained by PCR from the genomic DNA of GBS strain HN016 with primers (*spxA2-liaR*-F/R), and purification of the PCR products was done using the HiPure PCR pure Mini Kit (Magen, D2121-03). Purified DNA fragments (242 bp; 600 ng per reaction) were incubated with the purified LiaR protein (5, 10, and 15 μg, corresponding to DNA: LiaR mass ratios of approximately 1:8.3, 1:16.7, and 1:25, respectively) in binding buffer (20 mM Tris-HCl, pH 7.5, and 10% glycerol), and incubated for 30 min at 30 °C. A 226 bp internal DNA fragments lacking the predicted LiaR-binding motif and amplified using the primers QI-F/R, served as negative control at 600 ng per reaction. The mixtures were electrophoresed on 6% polyacrylamide gels in 1 × TBE buffer for 120 min at 100 V, stained with GelRed (MERCK, S9430) for 20 min, and visualized using a Vilber Lourmat E-Box VX5 imager.

### Growth curves

To assess growth under bile salt stress, mid-log phase GBS cultures (OD_600_ = 0.65) were adjusted to an OD_600_ = 0.2 in pre-warmed THB medium. 200 µL of the culture were transferred to a 96-well microtiter plate. The test groups contained THB supplemented with 0.01% (w/v) bile salts, while the control group contained THB only. The plate was incubated at 37°C in a microplate reader, and OD_600_ was measured every hour for 14 h.

For growth assays in the intestinal environment, intestinal contents were aseptically collected from the foregut of five healthy Nile tilapia (*Oreochromis niloticus*, 10 ± 1 g, sourced from Yueqiang Feng Hatchery, Guangdong, China) that were reared under standardized conditions and fasted for 24 h. One gram of the contents was homogenized in 1 mL of PBS (pH 7.0) using glass beads, and were harvested by centrifugation at 5,000 × *g* for 5 min to remove large debris and beads. The supernatant was filter sterilized using a 0.22 µm Millex^TM^-GP filter (MilliporeSigma, SLGP033R), and the sterility of the samples was confirmed by plating an aliquot on THB agar. The sterile intestinal content suspension was serially diluted in THB medium to final concentrations of 1:50 in a 96-well plate, with 100 µL per well (*n* = 3 technical replicates per dilution). Mid-log phase GBS cultures (OD_600_ = 0.65) were washed and adjusted in THB medium to an OD_600_ = 0.2. Then, 100 µL of each bacterial suspension was added to 100 µL of the intestinal content dilutions (final volume 200 µL/well). The negative control consisted of 100 µL of intestinal contents mixed with 100 µL of THB without bacterial inoculation. Bacterial growth was monitored as described above.

### *In vivo* survival and colonization assays

Healthy Nile tilapia were acclimated for 2 weeks prior to experimentation. For the survival study, 90 fish were randomly distributed into three groups (*n* = 30 per group). The fish were anaesthetized with 100 mg/L MS-222 (MCE; HY-W011777) and orally gavaged with 2.4 × 10^6^ CFU of GBS strain HN016 (WT) or Δ*spxA2* in a volume of 100 μL PBS, while control fish were gavaged with 100 μL of PBS. Mortality of tilapia was recorded on a daily basis for 14 d post-infection, and the dead fish was removed immediately.

For GBS colonization analysis, 20 fish were randomly allocated into two groups (*n* = 10 per group) and orally gavaged with 3.6 × 10^4^ CFU of GBS strain HN016 WT or Δ*spxA2* mutant in a volume of 100 μL PBS. At 3 d post-infection, three fish per group were euthanized using 400 mg/L MS-222 following the American Veterinary Medical Association’s guidelines and tissues (intestine, kidney, spleen, brain) were collected. Bacterial densities in tissues were quantified by homogenizing tissues, performing tenfold serial dilutions, and plating on THB agar plates. Following incubation for 16 h, viable bacteria were enumerated, and bacterial densities expressed as CFU/mL of tissue. All procedures were reviewed and approved by Animal Care and Use Committee of Huazhong Agriculture University (Approval No. HZAUFI-2024–0025). The experiments were conducted in accordance with the ARRIVE guidelines.

### Histopathological analysis

Healthy Nile tilapia were randomly assigned into three groups (*n* = 6 per group) and orally gavaged with 3.6 × 10^4^ CFU of GBS strain HN016 (WT) or Δ*spxA2* in a volume of 100 μL while control fish were orally gavaged with 100 μL of PBS. At 3 d post-infection, intestine tissues were collected and fixed in 4 % (w/v) paraformaldehyde (Solarbio, P1110) at room temperature for 24 h. Following fixing, the tissues were dehydrated through a graded ethanol series and embedded in paraffin. Serial sections of 4 μm thickness were prepared using a rotary microtome (Leica, RM2235) and stained with hematoxylin and eosin (H&E) for nuclear and cytoplasmic visualization, respectively. Histopathological analysis was performed using a Nikon Eclipse Ci microscope (Japan) and representative fields were photographed.

### Statistical analysis

Three independent experiments were performed, each with a single biological replicate. Data are presented as mean ± standard deviation (SD). Statistical analyses were conducted using GraphPad Prism version 9.0 (GraphPad Software, USA). Statistical differences between groups were analyzed using the Student’s *t*-test. For comparisons among multiple groups, one-way/two-way ANOVA followed by Tukey’s multiple-comparisons test was used. The Log-rank test was applied to assess differences in survival rates among groups in the tilapia infection assay. ****p < 0.0001; ***p < 0.001; **p < 0.01; *p < 0.05; ns, *p* > 0.05.

## Results

### SpxA2 plays a key role in bile salt resistance in GBS

**Figure 1.**
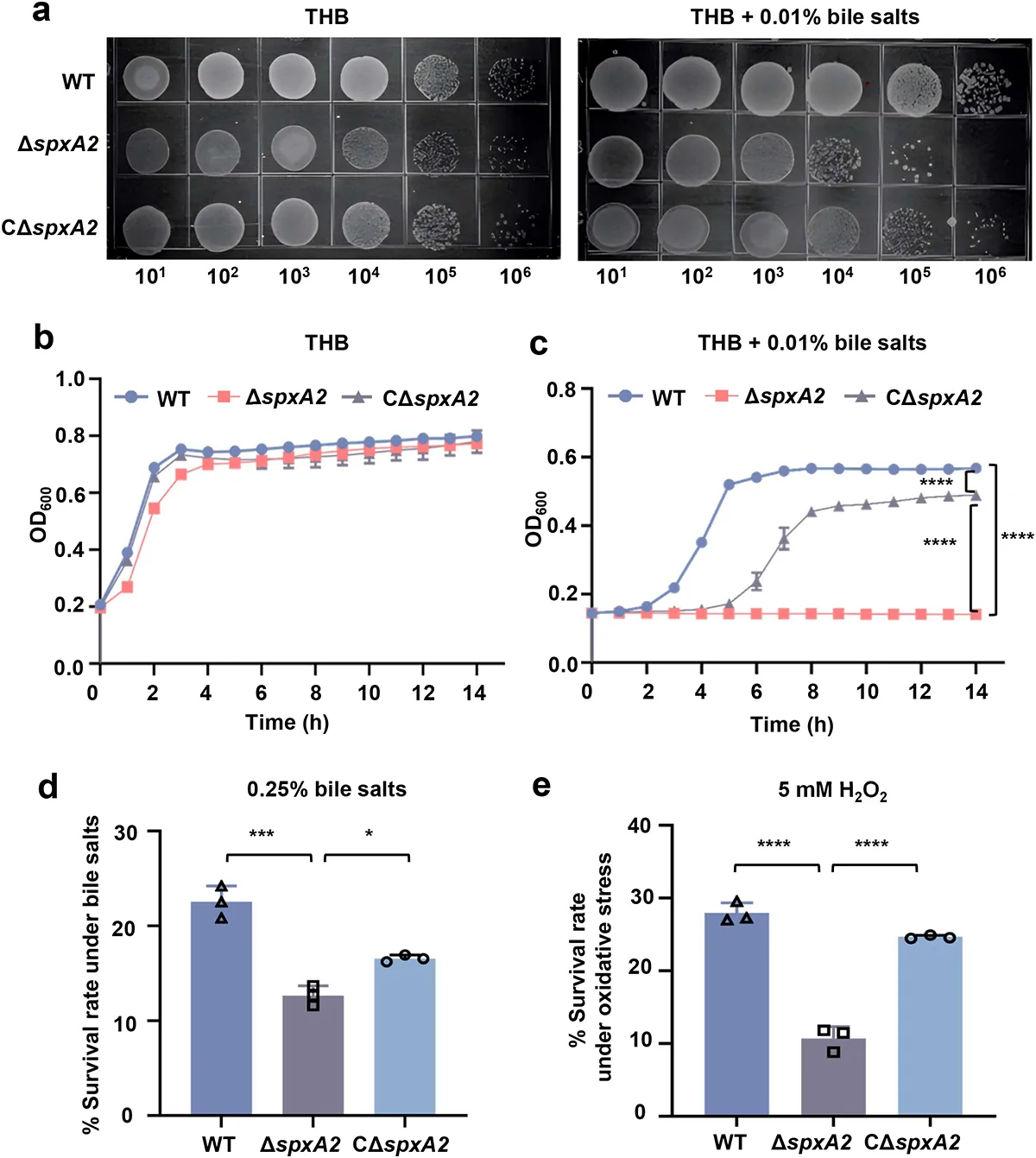
SpxA2 mediates bile salt tolerance in GBS. (a) Bile salt sensitivity of the Δ*spxA2* mutant. Spot dilution assays of GBS strain HN016 wild-type (WT), Δ*spxA2,* and CΔ*spxA2* strains on THB agar supplemented with or without 0.01% (w/v) bile salts. Tenfold serial dilutions of bacterial cultures were spotted onto the plates and incubated at 37 °C for 16 h. Bacterial growth of GBS WT, Δ*spxA2,* and CΔ*spxA2* strains in THB medium (b) or in THB medium containing 0.01% bile salts (c) were monitored by measuring the OD_600_ every hour for 14 h. (d) Survival rates under bile salt stress of the GBS WT, Δ*spxA2,* and CΔ*spxA2* strains. The strains in the mid-log phase (OD_600_ = 0.65) were exposed to 0.25% bile salts for 30 min. Bacterial mixtures were tenfold serially diluted and plated on THB agar. Bacterial viability was quantified by counting colony-forming units per milliliter (CFU/mL). The survival rate was expressed as the CFU count after treatment relative to the CFU count before treatment. (e) Survival rates under oxidative stress. The strains in the mid-log phase (OD_600_ = 0.65) were exposed to 5 mM H_2_O_2_ for 30 min. Bacterial viability was quantified as described above. The survival rate was expressed as the CFU count after treatment relative to the CFU count before treatment. Data are presented as the mean ± standard deviation from three independent biological replicates, each with a single biological replicate. Statistical difference was assessed using one-way ANOVA followed by Tukey’s multiple-comparisons test. Growth curves were analyzed using two-way ANOVA followed by Tukey’s multiple-comparison test (****p < 0.0001; *** *p* < 0.001; * *p* < 0.05).

To determine genes required for bile salt resistance in GBS, we generated a transposon mutant library in the background of GBS strain HN016. Initial screening of the Himar1 transposon mutant library on THB agar containing 0.015% bile salts identified six mutants with increased sensitivity to bile salts (Figure S3a and S3b; Table S3). Among these candidates, transposon mutant L34, which carries an insertion in SAHN016_RS09865 (Table S3), displayed marked bile salt hypersensitivity and was selected for further investigation. Sequence alignment indicated that SAHN016_RS09865 corresponds to *spxA2* (Figure S4a). To confirm the role of SpxA2 in bile salt resistance, the WT strain and the ∆*spxA2* and C∆*spxA2* mutants were cultured on THB agar plates with or without 0.01% bile salts. All three strains exhibited comparable growth on THB agar lacking bile salts. In contrast, the ∆*spxA2* mutant exhibited approximately a 1–2 log10 reduction in growth relative to the WT strain under bile salt stress. The complemented strain C∆*spxA2* partially restored this growth defect, although its growth remained slightly reduced compared with the WT strain at higher dilutions (Figure 1(a)). Consistent with these results, all three strains grew indistinguishably in THB medium without bile salts (Figure 1(b)). However, the ∆*spxA2* mutant completely failed to grow in THB medium containing 0.01% bile salts. The C∆*spxA2* strain substantially restored growth, although a delay was observed during the early growth phase compared to the WT strain (Figure 1(c)). qRT-PCR analysis showed that *spxA2* expression was markedly reduced in Δ*spxA2* mutant (*p* < 0.0001) and partially restored in CΔ*spxA2* (*p* < 0.01) compared with the WT strain (Figure S4b). Additionally, the survival rate of the ∆*spxA2* mutant was 12 ± 2%, which was significantly lower than that of 23 ± 2% in the WT strain (*p* < 0.001) and 16 ± 1% in the C∆*spxA2* strain (*p* < 0.05) after exposure to 0.25% bile salts for 30 min (Figure 1(d)). Moreover, the ∆*spxA2* mutant displayed a significantly reduced survival rate of 11 ± 2%, representing a significant marked decrease relative to the 28 ± 2% in the WT strain (*p* < 0.0001) and the 24 ± 1% in the C∆*spxA2* strain (*p* < 0.0001) under H_2_O_2_ exposure (Figure 1(e)). Altogether, these results demonstrate that SpxA2 plays a key role in bile salt and oxidative stress resistance in GBS.

### SpxA2 is required for maintaining membrane homeostasis in GBS

**Figure 2.**
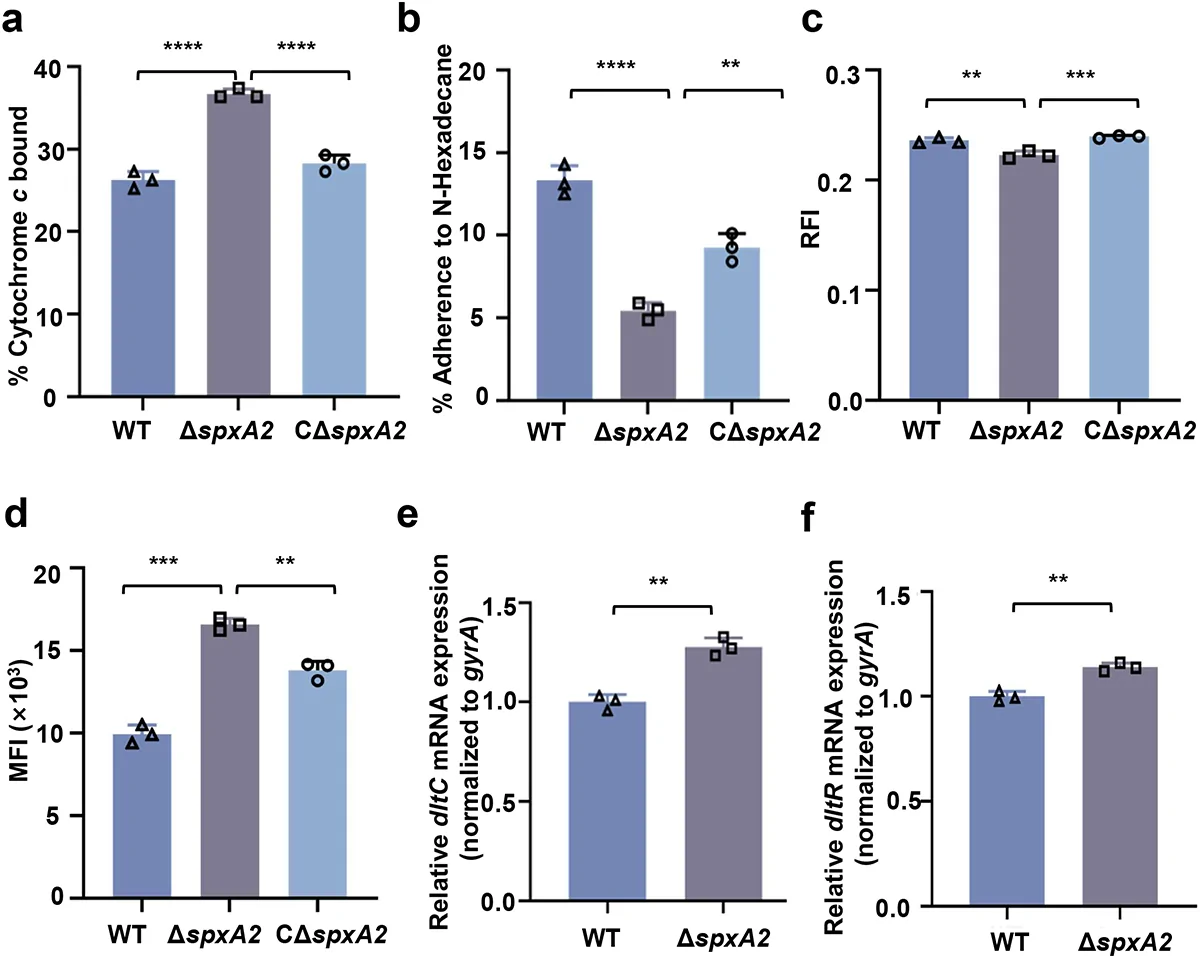
SpxA2 regulates membrane homeostasis in GBS. (a) Cytochrome *c* binding was used as an indicator of bacterial surface charge. GBS strain HN016 wild-type (WT), Δ*spxA2,* and CΔ*spxA2* mutants were incubated with cytochrome *c* at a final concentration of 0.2 mg/mL. The amount of unbound cytochrome *c* in the supernatant was quantified by measuring the absorbance at 530 nm. (b) Bacterial surface hydrophobicity. GBS WT, Δ*spxA2*, and CΔ*spxA2* strains were resuspended in PUM buffer and then mixing with N-hexadecane. Following phase separation, the OD_600_ of the lower aqueous phase was measured, and surface hydrophobicity was calculated as (1 − OD_600 aqueous_/OD_600_ initial) × 100%. (c) Bacterial membrane potential. GBS WT, Δ*spxA2,* and CΔ*spxA2* strains were exposed to 0.25% bile salts for 30 min, stained with DiOC_2_(3), and analyzed by flow cytometry. The median relative of fluorescence intensity (RFI) of red to green fluorescence was shown and used to assess bacterial membrane potential. (d) Bacterial membrane damage quantification. GBS WT, Δ*spxA2*, and CΔ*spxA2* were exposed to 0.25% bile salts for 30 min, stained with propidium iodide (PI), and fluorescence signals were measured by flow cytometry. The median fluorescence intensity (MFI) of PI staining was quantified to reflect membrane damage. Transcript levels of *dltC* (e) and *dltR* (f). Expression of *dltC* and *dltR* was quantified by qRT-PCR in GBS WT, Δ*spxA2* and CΔ*spxA2* strains and normalized to the housekeeping gene *gyrA*. Statistical difference was assessed using one-way ANOVA followed by Tukey’s multiple-comparisons test (a–d) and the unpaired Student’s *t*-test (e–f). Data are presented as the mean ± standard deviation from three independent biological replicates, each with a single biological replicate (****p < 0.0001; ***p < 0.001; **p < 0.01).

Bacterial surface charge, a key physicochemical parameter that influences nutrient uptake and stress resistance [47], was determined by cytochrome *c* binding assay. As shown in Figure 2(a), the ∆*spxA2* mutant exhibited a significantly higher level of cytochrome *c* adsorption (37 ± 2%) compared to the WT (26 ± 2%, *p* < 0.0001) and C∆*spxA2* (28 ± 2%, *p* < 0.0001) strains. This result indicates that the ∆*spxA2* mutant possesses an increased net negative surface charge. We next assessed whether the altered surface charge affected cell surface hydrophobicity using N-hexadecane. The ∆*spxA2* mutant exhibited a significantly lower affinity for N-hexadecane (5 ± 1%) compared to the WT (13 ± 2%, *p* < 0.0001) and C∆*spxA2* (9 ± 1%, *p* < 0.01) strains (Figure 2(b)), reflecting a decrease in cell surface hydrophobicity. Furthermore, DiOC_2_(3) was used to evaluate the membrane potential. The ∆*spxA2* mutant showed increased fluorescence in both red and green channels compared to the WT strain (Figure S5a-d). However, the relative fluorescence intensity (RFI) ratio was significantly decreased in the ∆*spxA2* mutant (0.222 ± 0.004) compared to the WT (0.235 ± 0.003, *p* < 0.01) and C∆*spxA2* strains (0.239 ± 0.002, *p* < 0.001) (Figure 2(c)), indicating membrane depolarization in the ∆*spxA2* mutant. Given the defect in membrane potential, we hypothesized that the ∆*spxA2* mutant might exhibit increased membrane permeability. Indeed, the ∆*spxA2* mutant showed significantly increased PI uptake compared to the WT strain (*p* < 0.0001) and the C∆*spxA2* mutant (*p* < 0.01) after 0.25% bile salt exposure for 30 min (Figure 2(d); Figure S5e and f), demonstrating enhanced membrane permeability in the ∆*spxA2* mutant. Enhanced membrane permeability might result in increased vulnerability to host immune responses during infection. To test this hypothesis, the intracellular survival of GBS strains within macrophages was examined. As shown in Figure S4c, the Δ*spxA2* mutant exhibited a significantly reduced intracellular survival in macrophages, decreasing to 47 ± 2% at 2 h and 35 ± 2% at 4 h post-infection, vs 72 ± 3% and 57 ± 3% for the WT strain (*p* < 0.0001 at both time points). The CΔ*spxA2* strain partially restored intracellular survival (57 ± 4% at 2 h and 43 ± 3% at 4 h post-infection), which was significantly higher than that of the Δ*spxA2* mutant at both time points (*p* < 0.01). Additionally, qRT-PCR analysis showed that the transcript levels of *dltC* and *dltR* were significantly increased in the Δ*spxA2* mutant compared with the WT strain (*p* < 0.01, Figure 2(e,f)). Collectively, these results demonstrate that SpxA2 plays a major role in membrane homeostasis in GBS.

### The LiaFSR system regulates s*pxA2* transcription

**Figure 3.**
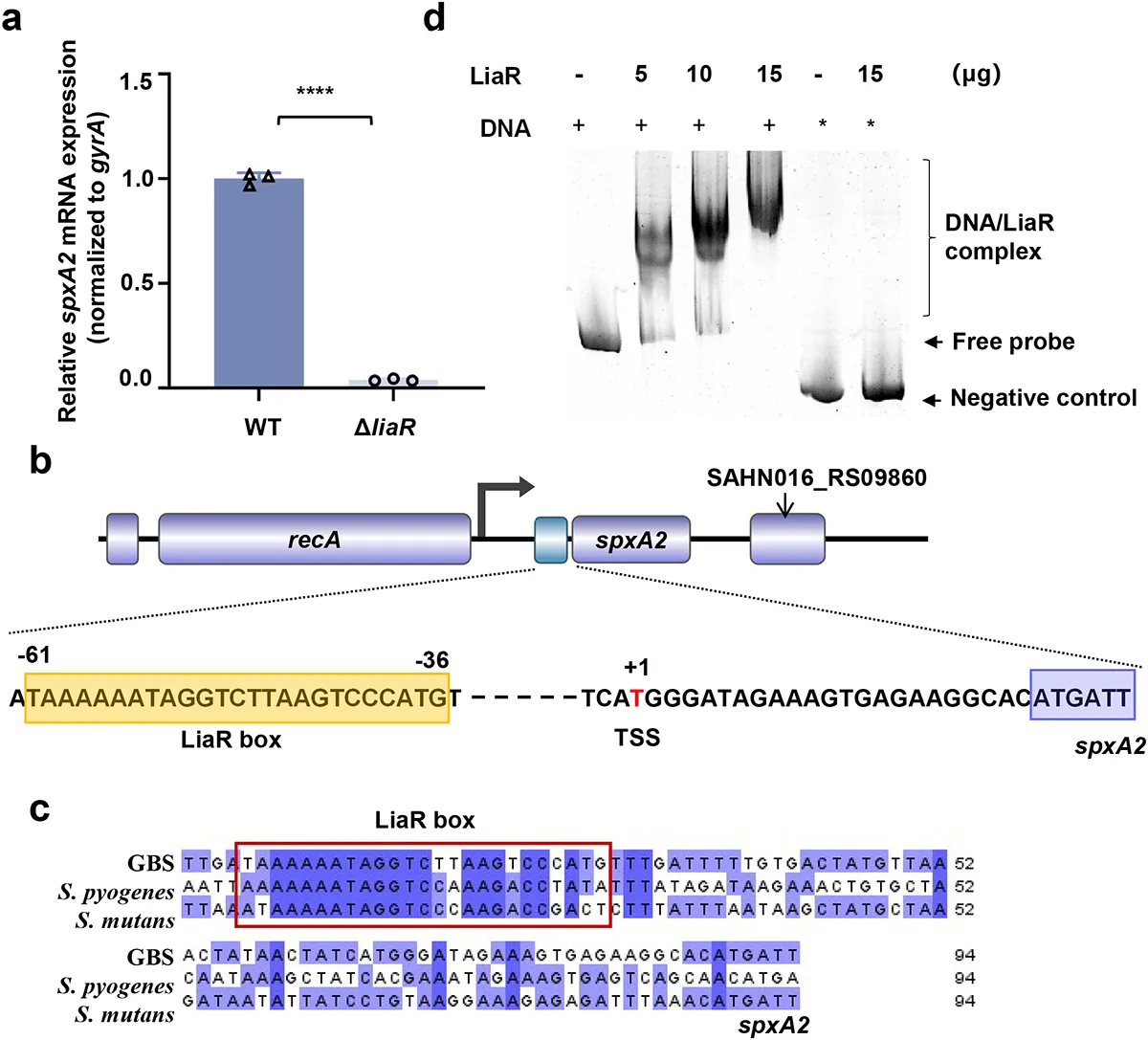
The LiaFSR system regulates *spxA2* expression. (a) Transcript levels of *spxA2* gene. Expression of *spxA2* was quantified by qRT-PCR in GBS wild-type (WT) and Δ*liaR* mutant, and normalized to the housekeeping gene *gyrA*. (b) Bioinformatic analysis of the *spxA2* promoter. Numbering is relative to the transcriptional start site (TSS, +1). The LiaR box is located at position from −36 to −61 relative to the TSS. (c) Multiple sequence alignment of *spxA2* promoter region across streptococci. DNA sequence alignment of the promoter region upstream of the *spxA2* gene in GBS strain HN016, *S. pyogenes* strain MGAS10870, and *S. mutans* strain UA159. The consensus LiaR-binding box is highlighted in the red box. The identical residues across all three sequences are highlighted in a dark blue, residues conserved or shared by two sequences are highlighted in light blue, and non-conserved residues are shown on a white background. (d) Electrophoretic mobility shift assay (EMSA) for LiaR binding with the *spxA2* promoter. EMSA showing the binding of purified LiaR protein to the *spxA2* promoter fragment. Each reaction contained 600 ng of the *spxA2* promoter fragment and 5, 10, or 15 μg of purified LiaR protein, corresponding to DNA: LiaR mass ratios of approximately 1:8.3, 1:16.7, and 1:25, respectively. Lanes “−:” no LiaR protein; “+:” *spxA2* promoter fragment; “*:” no LiaR specific binding motif DNA. Free probe: free DNA. The free probe and DNA/LiaR complex are indicated. The unpaired Student’s *t*-test was applied for data analysis. Data are presented as the mean ± standard deviation from three independent replicates, each with a single biological replicate (****p < 0.0001).

Given that SpxA2 is a cytosolic transcriptional regulator and that its expression is conserved and regulated by the LiaFSR system [29], we next investigated whether LiaR, the response regulator of the LiaFSR TCS involved in the cell envelope stress response, contributes to the regulation of *spxA2* expression in GBS. Consistent with this hypothesis, the *spxA2* transcription levels were significantly reduced in the Δ*liaR* mutant compared to the WT strain (*p* < 0.0001; Figure 3(a)). To assess whether this regulation was direct, we analyzed the *spxA2* promoter region for a potential LiaR binding site. Sequence alignment using MEME revealed a conserved LiaR binding motif located from −61 to −36 bp upstream of the transcriptional start site (TSS) of the *spxA2* gene (Figure 3(b)). Cross-species promoter alignment further showed that this putative LiaR binding site is conserved between GBS, *S. pyogenes* and *S. mutans* (Figure 3(c)). To determine whether LiaR directly interacts with *spxA2* promoter, an EMSA was carried out. Recombinant LiaR protein produced a clear mobility shift of the *spxA2* promoter fragment (Figure 3(d)), indicating direct binding. The specificity of this interaction was demonstrated by the absence of binding to a control DNA fragment lacking the predicted LiaR motif (Figure 3(d)). Together, these findings indicate that LiaR directly interacts with the *spxA2* promoter region and activates its transcription.

### The LiaFSR system is vital to the bile salt resistance in GBS

**Figure 4.**
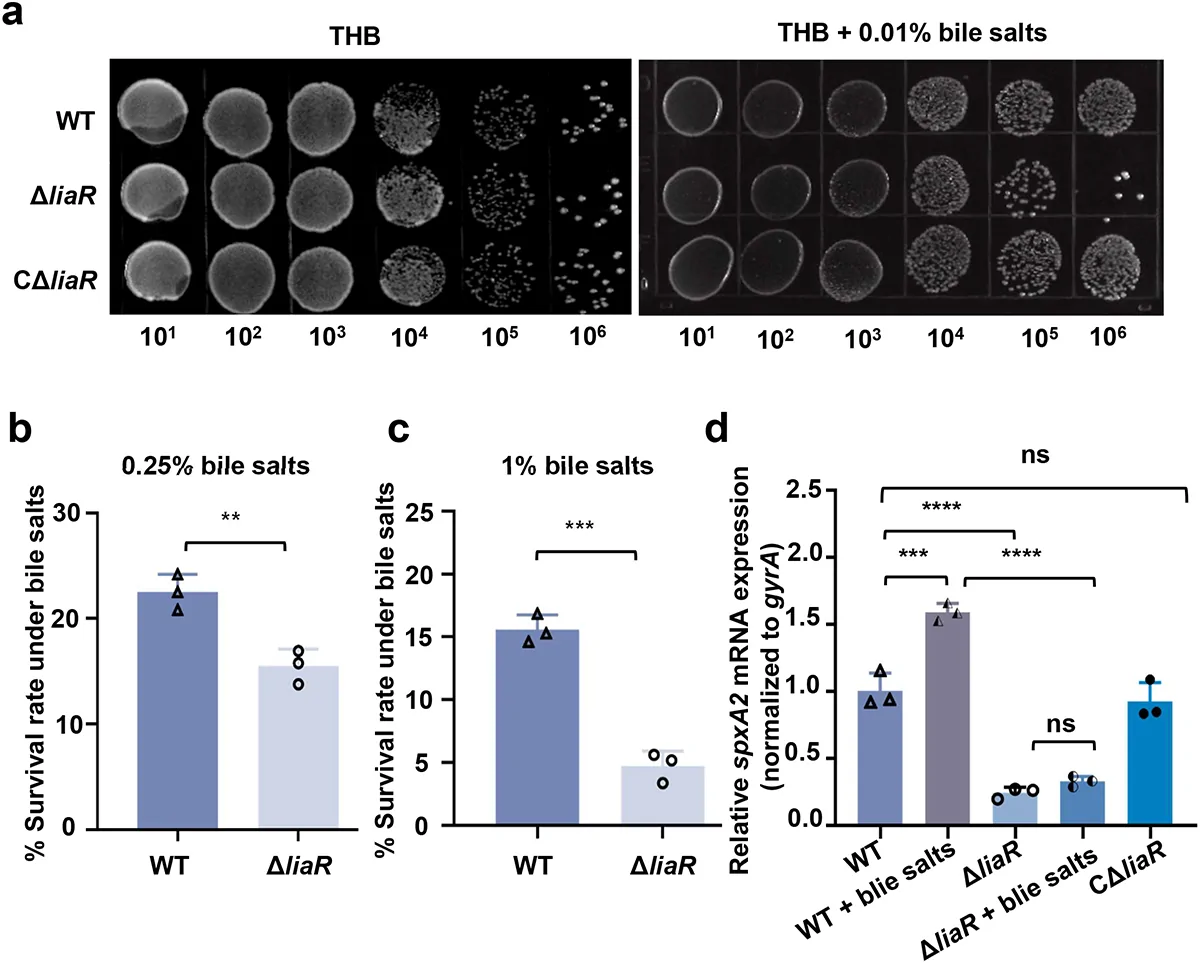
The LiaFSR system mediates bile salt tolerance in GBS. (a) Bile salt sensitivity of GBS strain HN016 (WT) and the Δ*liaR* and CΔ*liaR* mutants on THB agar with or without 0.01% (w/v) bile salts. Tenfold serial dilutions of bacterial cultures were spotted onto the plates and incubated at 37°C for 16 h. Survival rates of GBS WT and the Δ*liaR* mutant after exposure to 0.25% (b) and 1% (c) bile salts for 30 min. Bacterial viability was quantified by counting colony-forming units per milliliter (CFU/mL). The survival rate was expressed as the CFU count after treatment relative to the CFU count before treatment. (d) Expression of *spxA2* in GBS WT, Δ*liaR,* and CΔ*liaR* strains with or without 0.25% bile salt challenge for 30 min, was quantified by qRT-PCR and normalized to the housekeeping gene *gyrA*. Data were analyzed using unpaired two-tailed Student’s *t*-test for (b, c) and one-way ANOVA followed by Tukey’s multiple-comparisons test for (d). Data are presented as the mean ± standard deviation from three independent biological replicates, each with a single biological replicate (****p < 0.0001; ***p < 0.001; **p < 0.01; ns, *p* > 0.05, ns: not significant).

Given that SpxA2 is critical for bile salt resistance and is directly activated by LiaR, we next evaluated the contribution of the LiaFSR system in bile salt resistance. The Δ*liaR* mutant exhibited markedly impaired growth on THB agar (Figure 4(a)) and in THB medium (Figure S6a) containing 0.01% bile salts, despite growing comparably to the WT strain under non-stress conditions on THB agar (Figure 4(a)) and in THB medium (Figure S6b). Additionally, the survival rate of the Δ*liaR* mutant was significantly lower than that of the WT strain following exposure to 0.25% bile salts at 15 ± 2% and 22 ± 2%, respectively (*p* < 0.01, Figure 4(b)). This sensitivity was even more pronounced at a higher concentration of 1% bile salts, where the survival rate of the Δ*liaR* mutant was significantly reduced to only 4 ± 2% vs 15 ± 2% for the WT strain (*p* < 0.001, Figure 4(c)). We then investigated the molecular mechanism underlying this phenotype by assessing *spxA2* expression. Upon exposure to 0.25% bile salts, the expression of *spxA2* was strongly induced in the WT strain (*p <* 0.0001; Figure 4(d)). However, this induction was abolished in the Δ*liaR* mutant, in which *spxA2* expression did not change significantly following bile salt exposure (*p* > 0.05; Figure 4(d)). Furthermore, under non-stressed conditions, *spxA2* expression in the Δ*liaR* mutant was significantly lower than in the WT (*p* < 0.0001; Figure 4(d)). This reduction was restored in the complemented CΔ*liaR* strain, where *spxA2* expression levels were comparable to those of the WT strain (*p* > 0.05; Figure 4(d)). In summary, these findings reveal a LiaR-SpxA2 regulatory axis in GBS, in which LiaR is required for the induction of *spxA2* expression in response to bile salt stress. The impaired induction of *spxA2* in the Δ*liaR* mutant provides a mechanistic link between disruption of this regulatory axis and the bile salt-sensitive phenotype. These results suggest that the LiaFSR system plays a key role in bile salt detection and resistance in GBS.

### SpxA2 is important for GBS fitness in the intestinal environment

**Figure 5.**
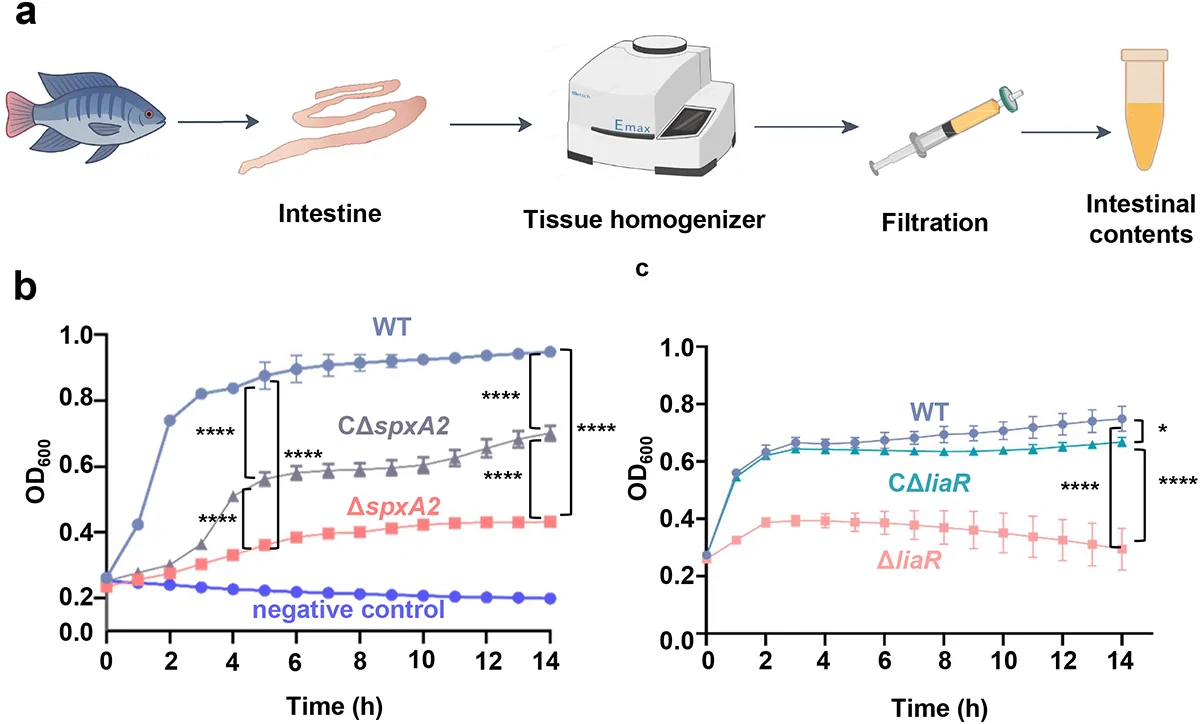
SpxA2 is required for GBS survival in the intestinal environment. (a) Schematic of *ex vivo* intestinal contents preparation. Intestines were aseptically collected from tilapia, and intestinal contents were homogenized in PBS using a tissue homogenization. The homogenate was then filtered to obtain intestinal content filtrates for subsequent assays. Bacterial growth in intestinal contents. Growth of GBS strain HN016 wild-type (WT), Δ*spxA2*, and CΔ*spxA2* mutants (b) and WT, Δ*liaR*, and CΔ*liaR* strains (c) in THB medium supplemented with 50% (w/v) sterile intestinal content suspension was monitored by measuring OD_600_ every hour for 14 h. The negative control consisted of 100 µL of intestinal contents mixed with 100 µL of THB. Growth curves were analyzed using two-way ANOVA followed by Tukey’s multiple-comparison test. Data are presented as the mean ± standard deviation from three independent biological replicates, each with a single biological replicate (****p < 0.0001; *p < 0.05).

Based on the observed impaired growth of the ∆*spxA2* mutant under bile salt stress, we hypothesized that its fitness would be compromised within the complex intestinal environment. We thus assessed bacterial growth in *ex vivo* tilapia intestinal contents (Figure 5(a)). The ∆*spxA2* mutant displayed markedly impaired growth in the presence of intestinal contents, achieving a final OD_600_ (0.4 ± 0.01) at 14 h post-inoculation that was significantly lower than that of both the WT (0.9 ± 0.01) and C∆*spxA2* strains (0.7 ± 0.02, *p* < 0.0001, Figure 5(b)). The complemented CΔ*spxA2* strain partially restored this growth defect, although its growth remained significantly reduced compared with the WT strain at 14 h post-inoculation. The Δ*liaR* mutant also showed significant reduced growth in intestinal contents compared with the WT strain, whereas the complemented CΔ*liaR* strain showed growth approaching that of the WT strain (Figure 5(c)). This demonstrates that SpxA2 is important for GBS to proliferate effectively in the presence of intestinal contents, underscoring its major role for bacterial fitness in the gut environment.

### SpxA2 contributes to GBS virulence *in vivo*

**Figure 6.**
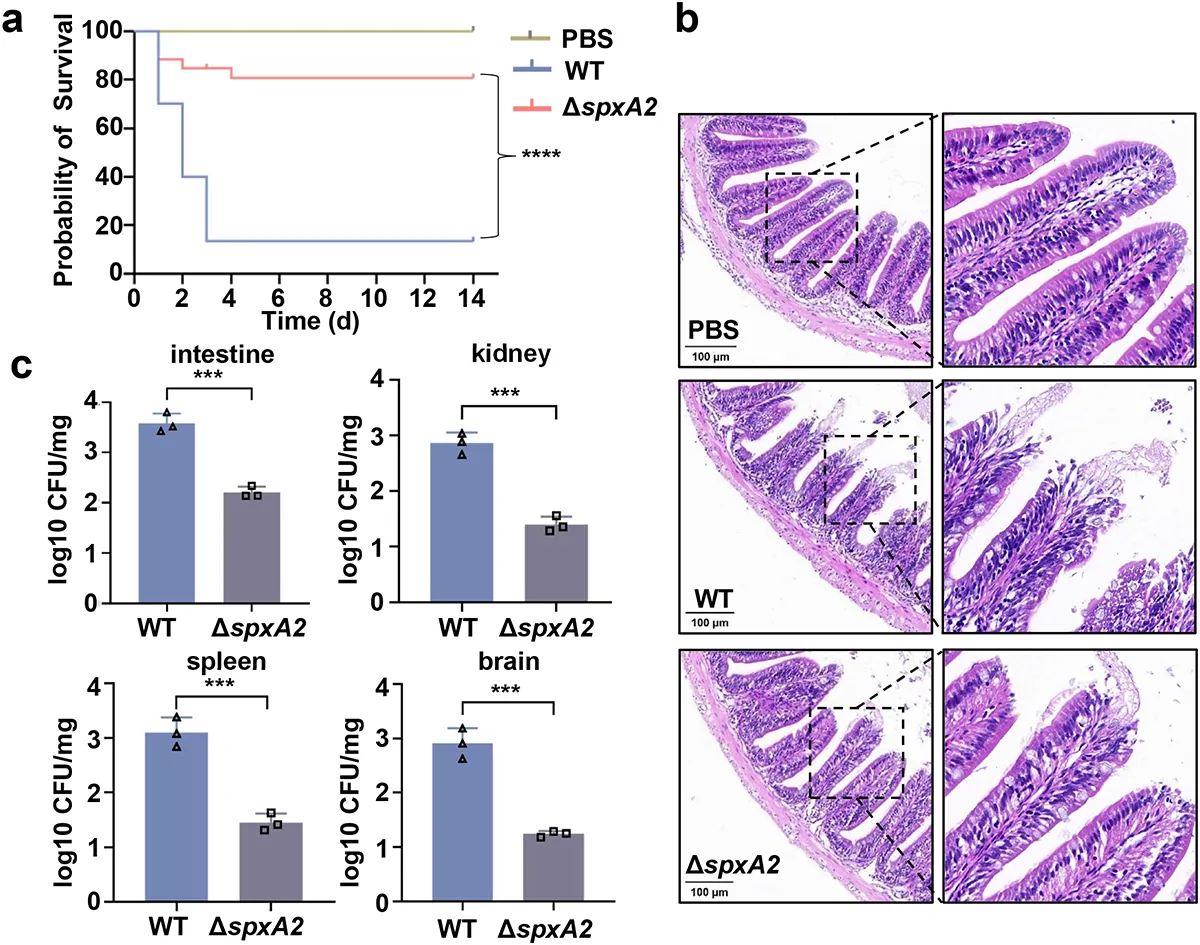
SpxA2 contributes to the pathogenicity in *vivo*. (a) In *vivo* survival assay. Tilapia (*n* = 30 per group) were orally gavaged with 2.4 × 10^6^ CFU (100 μL) of GBS strain HN016 wild-type (WT) or Δ*spxA2* mutant, or with 100 μL of PBS as a control. Survival was monitored daily for 14 d post-infection. (b) Histopathological assessment of intestinal damage. Representative hematoxylin and eosin (H&E)-stained sections of intestinal tissues illustrating tissue architecture and pathological alterations following infection. (c) Bacterial colonization in host tissues. Bacterial loads in intestine, kidney, spleen, and brain of infected tilapia were determined at 3 d post-infection. The log-rank test was applied to assess differences in survival rates between groups. The unpaired Student’s *t*-test was applied for data analysis. Data are presented as the mean ± standard deviation from three independent biological replicates, each with a single biological replicate (****p < 0.0001; ***p < 0.001).

To evaluate the contribution of SpxA2 in GBS pathogenesis, tilapia were orally gavaged with 100 μL of WT or ∆*spxA2* strains at 2.4 × 10^6^ CFU per fish. Control fish were gavaged with the same volume of PBS, and the survival was monitored for 14 d. The WT-infected group exhibited severe mortality, with a final survival rate of only 13.3%. In contrast, fish infected with the Δ*spxA2* mutant showed a significantly higher survival rate of 83.3% (*p* < 0.0001 vs WT; Figure 6(a)), while all fish in the PBS-control group survived the infection. Histopathological examination of intestinal tissues demonstrated extensive disruption of intestinal villi in WT-infected fish, whereas the ∆*spxA2*-infected fish showed minor alterations in the intestinal villi, indicating an attenuated pathological effect (Figure 6(b)). We also quantified the bacterial density in major organs at 3 d post-infection. Compared to the WT strain, the Δ*spxA2* mutant was characterized by significantly reduced bacterial loads in intestine (*p* < 0.001), kidney (*p* < 0.001), spleen (*p* < 0.001) and brain (*p* < 0.001) (Figure 6(c)). Collectively, these findings confirm that SpxA2 contributes to the virulence and tissue dissemination of GBS.

## Discussion

GBS is considered an important conditional pathogenic bacterium that has the ability to establish colonization on host’s intestinal epithelial cells [12,48,49]. Bile salts, as key antimicrobial components in the gut environment, disrupt bacterial cell membranes [17,49], induce oxidative stress, cause protein misfolding, and lead to DNA damage. Thus, bile salt sensing and adaptation are essential for the survival of intestinal microbes. However, the genetic determinants that enable GBS to withstand this challenge remain incompletely characterized. To address this, we constructed a transposon mutant library in the GBS strain HN016 and identified a mutant with an insertion in the *spxA2* gene resulting in a marked hypersensitivity to bile salts. We selected SpxA2 for further investigation because it is conserved among Firmicutes, including notable human pathogens such as *Bacillus anthracis*, *Staphylococcus aureus*, *Enterococcus faecalis*, *Streptococcus* spp., and *Listeria monocytogenes* and it represents a promising candidate for orchestrating a global adaptive response to intestinal stress. While SpxA2 has previously been implicated in thermal [50] and antibiotic [29] stress responses in other bacterial species, its function in GBS, particularly in the context of bile salt resistance, was unknown. Our findings identified a previously uncharacterized role for SpxA2 in bile salt resistance, as demonstrated by the pronounced growth defect of the Δ*spxA2* mutant under bile salt stress and the partial restoration of bile salt resistance in the CΔ*spxA2* mutant. The incomplete restoration to WT levels in some assays may be partly explained by the lower *spxA2* transcript level in the CΔ*spxA2* mutant and the expression of *spxA2* from a constitutive *lac* promoter rather than the native regulatory context. Because GBS strain HN016 encodes two Spx homologs, we sought to distinguish the bile salt response of SpxA1 from that of SpxA2. Whole genome sequence alignment revealed that the sequence SAHN016_RS05130 corresponds to SpxA1, whereas the characterized *spx* in this study corresponds to SpxA2 (SAHN016_RS09865). qRT-PCR analysis showed that the *spxA1* transcription was downregulated in ∆*liaR* mutant but unaffected by bile salt exposure in the WT and Δ*spxA2* strains (Figure S4d). By contrast, *spxA2* expression was strongly induced in the WT and also significantly upregulated in two additional GBS strains, COH1 and A909, upon bile salt challenge (Figure S4e). These data suggest that the bile salt resistance phenotype is primarily mediated by SpxA2 in GBS.

The increased negative surface charge of the Δ*spxA2* mutant was observed, which was correlated with enhanced cell surface hydrophilicity. These interrelated physicochemical alterations are consistent with a fundamental change in cell wall architecture, as charge and hydrophobicity are governed by surface-exposed molecules [51]. Teichoic acids are key components of the bacterial cell wall that contribute to the surface charge in most Gram-positive bacteria [52], and their Dalanylation, mediated by the *dlt* operon, reduces net negative surface charge while maintaining cell envelope integrity [53]. Given that SpxA2 is a conserved global cytosolic transcriptional regulator in Bacillota that activates gene expression by binding to the C terminal domain of the RNA polymerase α subunit (RNAP αCTD) and target promoters [54], we initially hypothesized that the increased negative surface charge of the Δ*spxA2* mutant was correlated with reduced *dlt* operon expression. However, qRT-PCR analysis revealed that *dltC* and *dltR* transcript levels were significantly increased in the Δ*spxA2* mutant, indicating that the increased negative charge could not be explained by decreased D-alanylation gene expression. This unexpected finding suggests that SpxA2 may influence surface charge through alternative mechanisms. As a cytosolic transcriptional regulator, SpxA2 is unlikely to directly interact with the bacterial membrane; rather, it may indirectly modulate membrane properties through transcriptional regulation of genes involved in cell envelope homeostasis.

In *S. mutans*, SpxA2 relays cell envelope stress signals from LiaR to downstream effectors that maintain cell wall and membrane homeostasis, including membrane fatty acid composition and ATPase activity [29]. Similarly, in *S. pyogenes*, the LiaFSR system responds to antimicrobial peptide-induced membrane stress by activating *spxA2* expression via phosphorylated LiaR, and that disruption of this regulatory axis leads to increased antimicrobial susceptibility and attenuated virulence [31,55]. Consistent with these reports, our study reveals that in GBS, exposure to bile salts activates the LiaFSR system, leading to LiaR-dependent upregulation of *spxA2*, which is required for maintaining membrane potential and integrity under bile salt stress. These findings collectively support a conserved role for the LiaFSR-SpxA2 regulatory axis in mediating cell envelope stress responses across pathogenic streptococci, while also revealing species-specific adaptations, such as the role of this system in bile salt resistance and intestinal colonization in GBS, which has not been reported in *S. mutans* or *S. pyogenes* [29,31,55]. Consistent with the disturbed membrane homeostasis phenotype, the Δ*spxA2* mutant also showed increased membrane permeability. The compromised membrane integrity likely facilitates the killing of the Δ*spxA2* mutant within macrophages, potentially by making it more vulnerable to antimicrobial peptides, reactive oxygen species, or other degradative components within the phagosome [56], thereby accounting for the severe intracellular survival defect. This hypothesis is further supported by the increased sensitivity of the Δ*spxA2* mutant to oxidative stress.

The LiaFSR-SpxA2 module is known to mediate canonical cell envelope stress responses to antibiotics and antimicrobial peptides in streptococci [30–33]. Although LiaFSR has been implicated in bile salt resistance in *Enterococcus faecium* [57,58], the role of this conserved pathway in streptococcal bile salt adaption has never been established. Our results demonstrated that the LiaFSR-SpxA2 is essential for mediating the response to bile salt challenge in GBS, as deletion of *liaR* or *spxA2* severely impaired bacterial survival under bile salt stress. The reduced basal expression of *spxA2* in the Δ*liaR* mutant in the absence of bile salts, a pattern also observed in *S. mutans* [29], suggests that LiaR may contribute to the maintenance of basal *spxA2* transcription under non-stress conditions. This observation raises the possibility that LiaR plays a broader role in *spxA2* regulation beyond stress-inducible activation. The residual SpxA2 signal is consistent with the possibility that SpxA2 abundance is influenced by additional regulatory mechanisms beyond LiaR, as YjbH has been shown to promote delivery of Spx to ClpXP for degradation in related Gram-positive bacteria [59,60]. These findings extend the functional role of the conserved regulatory axis LiaFSR-SpxA2 in streptococci beyond antibiotic and antimicrobial peptide resistance to encompass adaption to the unique bile salt‑rich environment in host intestine.

Given the role of bile salts as natural intestinal antimicrobial, impaired bile salt resistance would be expected to compromise bacterial colonization in host intestine. Indeed, the Δ*spxA2* mutant showed significantly reduced survival in tilapia intestinal contents, indicating that SpxA2 is critical for GBS persistence in the gut environment. To assess the physiological relevance of these findings, we employed a well-established tilapia oral infection model in which the Δ*spxA2* mutant exhibited attenuated lethality, reduced bacterial loads in tissue, and diminished intestinal epithelial damage compared to the WT strain. This attenuated virulence aligns with previous reports that Spx proteins regulate stress responses and virulence in *Streptococcus mutans* [28], supporting the conserved role of SpxA2 in streptococcal virulence.

In summary, this study defines an important LiaFSR-Spx regulatory axis that is essential for bile salt resistance in GBS. We further show that this axis is critical for GBS survival in the intestinal environment and systemic pathogenesis *in vivo* model. Our findings support a model in which the preservation of cell membrane integrity by the LiaFSR-SpxA2 pathway enables GBS to overcome biliary defenses in the gut, thereby facilitating intestinal colonization, dissemination, and subsequent infection.

## Supplementary Material

Supplemental Material

Supplementary_Material (1)clean.docx

## Data Availability

The HN016 genomic DNA sequence data is available in the NCBI GenBank database (NZ_CP011325). All data supporting this study are available in figshare at https://doi.org/10.6084/m9.figshare.30620732 [61].
