# Supplementary material for: The LiaFSR-SpxA2 regulatory axis governs bile salt resistance and virulence in Group B Streptococcus
Source: Virulence. 2026 Aug 19;17(1):2721814. doi: 10.1080/21505594.2026.2721814 (PMC13531978; doi:10.1080/21505594.2026.2721814)
Supplement: Supplementary file 1 [file KVIR_A_2721814_SM2301.docx]

**Figure S1: Genotypic verification of the ∆*spxA2* and ∆*liaR*.** (a) PCR analysis of ∆*spxA2.* The Lanes 1 and 2 represent the amplification of the *spxA2* gene using the primers *spxA2-*exterior-F/R. Lanes 3 and 4 represent the amplification of the *spxA2* gene border using the primers *spxA2-*interior-F/R. Lanes 1 and 3 use genomic DNA of GBS strain HN016 as template, Lanes 2 and 4 use genomic DNA of ∆ *spxA2* as template. (b) Lanes 1, 2 and 3 represent the amplification of the *liaR* gene using the primers *liaR-*exterior-F/R. Lanes 4, 5 and 6 represent the amplification of the *liaR* gene border using the primers *liaR-*interior-F/R. Lanes 3 and 6 use genomic DNA of HN016 as template, Lanes 1, 2 and 4, 5 use genomic DNA of ∆*liaR* as template. PCR products were analyzed by electrophoresis on a 1% agarose gel. Marker : DL2000 (Takara).

**Figure S2: Analysis of the purified LiaR protein.** (a) SDS-PAGE analysis of LiaR during purification*.* Proteins were separated by SDS-PAGE and visualized by coomassie brilliant blue staining. Lane M: 180 kDa Prestained Protein Marker (Vazyme; MP102-01); Lane 1: the total lysis of *E. coli* BL21 cells carrying pET28a-*liaR*; Lane 2: the flow through fraction; Lanes 3–9 represent elution fractions obtained with different concentrations of imidazole: lane 3, 50 mM; lane 4, 100 mM; lanes 5–7, 200 mM; lane 8, 400 mM; and lane 9, 500 mM. The arrow indicates the purified LiaR protein. (b) Western-blotting analysis of the purified LiaR protein. The protein from lane 8 shown in (a) was probed with an His-tag antibody (1:2000 dilution) to confirm the identity of the His-tagged LiaR protein.

**Figure S3: Screening of the GBS transposon mutant library for altered bile salts tolerance.** Primary screening for bile salts sensitivity (a and b). The Himar1 transposon mutant library was plated on THB agar supplemented with 0.015% (w/v) bile salts. Putative bile salts sensitive mutants exhibiting reduced growth are indicated by circles. The *spxA2* transposon insertion mutant is highlighted with an arrow.

**Figure S4: Conservation of SpxA2 in Streptococci and characterization of the Δ*spxA2* mutant and complemented strain.** (a) Multiple sequence alignment of SpxA2 homologs. Amino acid sequences of SpxA2 from GBS strain HN016 wild-type (WT), GBS strain A909, *S. suis* strain 3112, *S. mutans* strain UA159, and *S. pyogenes* strain MGAS10870 were aligned. The identical residues across all sequences are highlighted in a dark blue, residues conserved or shared by at least two sequences are highlighted in light blue, and non-conserved residues are shown on a white background. (b) Transcript levels of *spxA2* gene. Expression of *spxA2* was quantified by qRT-PCR in GBS WT, Δ*spxA2* and CΔ*spxA2* strains, and normalized to the housekeeping gene *gyrA.* (c) Intracellular survival in macrophages. RAW264.7 macrophages were infected with GBS WT, Δ*spxA2*, or CΔ*spxA2* at an MOI = 10:1. After infection, extracellular bacteria were eliminated with gentamicin and penicillin G, and intracellular bacteria were recovered from macrophage lysates at 0, 2, and 4 h post-infection. Survival rates were calculated as CFU/mL at each time point relative to CFU/mL at 0 h. (d) Transcript levels of *spxA1* gene under bile salts stress. Expression of *spxA1* gene in WT, Δ*spxA2,* Δ*liaR* strains with or without 0.25% bile salt challenge was quantified by qRT-PCR and normalized to the housekeeping gene *gyrA*. (e) Transcript levels of *spxA2* gene in GBS HN016, COH1 and A909 strains under bile salts stress. Expression of *spxA2* gene in GBS strains with or without 0.25% bile salt challenge was quantified by qRT-PCR and normalized to the housekeeping gene *gyrA*. Statistical differences were determined using the ordinary one-way ANOVA followed by Tukey’s multiple-comparisons test (b) and using the two-way ANOVA followed by Tukey’s multiple-comparisons test (c, d). Data are presented as the mean ± standard deviation from three independent biological replicates. (*****p* < 0.0001; ***p* < 0.01, ns: not significant.)

**Figure S5: Representative flow cytometry histograms of DiOC2(3) fluorescence and PI uptake in GBS WT, Δ*spxA2*, and C*ΔspxA2* strains following exposure to 0.25% bile salts for 30 min.** Analysis of membrane potential using DiOC2(3) staining. (a) and (b) show DiOC2(3) fluorescence detected in the green (FITC) channel, whereas (c) and (d) show DiOC2(3) fluorescence detected in the red (PE) channel. The red/green fluorescence ratio was calculated from the PE and FITC fluorescence signals and used to evaluate membrane potential. Analysis of membrane potential using PI staining. (e) and (f) show PI staining detected in the PE channel to assess membrane permeability. (a), (c), and (e) show the corresponding unstained negative controls, and (b), (d), and (f) show the stained samples. The y-axis shows event counts.

**Figure S6:** **Growth kinetics of GBS WT, Δ*liaR* and CΔ*liaR* strains.** Growth curves of different strains in THB medium without (a) or with 0.01% bile salts (b) were monitored by measuring the OD_600_ every hour for 14 h.
