# Supplementary material for: The LiaFSR-SpxA2 regulatory axis governs bile salt resistance and virulence in Group B Streptococcus
Source: Virulence. 2026 Aug 19;17(1):2721814. doi: 10.1080/21505594.2026.2721814 (PMC13531978; doi:10.1080/21505594.2026.2721814)
Supplement: Supplementary file 2 [file KVIR_A_2721814_SM5070.docx]

**Table S1 Summary table of strains and plasmids used in this study**

| **Strain and plasmid** | **Characteristics and function** | **Sources** |
| --- | --- | --- |
| **GBS strains** |  |  |
| HN016 | the wild type virulent GBS isolated from tilapia with meningoencephalitis, China | Collected in our laboratory |
| ∆*spxA2* | *spxA2* gene deletion mutant in HN016 background | This study |
| C∆*spxA2* | Complemented ∆*spxA2* strain (C∆*spx*); Spc^R^ | This study |
| ∆*liaR* | *liaR* gene deletion mutant in HN016 background | This study |
| ***E. coli* strains** |  |  |
| *E. coli* DH5α | Cloning host for plasmid propagation | Vazyme, China |
| *E. coli* BL21(DE3) | Host for expressing recombinant proteins | Vazyme, China |
| **Plasmids** |  |  |
| pSET4s | Temperature-sensitive suicide vector for gene knockout in *S. agalactiae*; Spcᴿ | Vazyme, China |
| pSET2 | *E. coli–Streptococcus* shuttle vector for genetic complementation; Spc^R^ | Vazyme, China |
| pET-28a | Plasmid vector for His-fused protein expression; Kan^R^ | Vazyme, China |
| pSET4s-Δ*spxA2* | Derived from pSET4s, carrying the Δ*spxA2* deletion fragment; Spcᴿ | This study |
| pSET4s-Δ*liaR* | Derived from pSET4s, carrying the Δ*liaR* deletion fragment; Spcᴿ | This study |
| pSET2- *spxA2* | Derived from pSET2, carrying the *spxA2* gene, for complementation of Δ*spxA2* strain; Spc^R^ | This study |
| pSET2- *liaR* | Derived from pSET2, carrying the *liaR* gene, for complementation of Δ*liaR* strain; Spc^R^ | This study |
| pET-28a-LiaR | Derived from pET-28a, carrying the *liaR* gene, for expressing LiaR protein; Kan^R^ | This study |

Note: Spc^R^, spectinomycin resistance; Kan^R^, kanamycin resistance.

**Table S2 Primers used in this study**

| **Primers** | **Sequences (5′→ 3′)** | **Application** |
| --- | --- | --- |
| M13-F | GTAAAACGACGGCCAGT | Verification of pSET4s recombinant plasmid construction (colony PCR/sequencing) |
| M13-R | CAGGAAACAGCTATGAC |  |
| *spxA2*-interior-F | GCACAAGTTGTAAAAAAGC | Internal verification of Δ*spxA2* mutant strain |
| *spxA2*-interior-R | GACGTTTGTCATCAATTAG |  |
| *spxA2*-exterior-F | ATCAACCAGCTAAGGG | External verification of Δ*spxA2* mutant strain |
| *spxA2*-exterior- R | CTAGCATACGCTCTGC |  |
| 4s-up-*spxA2*-F | GTGACTCTCACTTATGCCTCACTTTCTATCCCATGATAG | Upstream homology arm amplification for *spxA2* gene deletion |
| 4s-up-*spxA2*-R | ACGCCAAGCTTGCATGCAGTAGGCGTTATGTTTGG |  |
| 4s-down-*spxA2*-F | ACGACGGCCAGTGAATTCCAGTCGTTAGACGTTCATC | Downstream homology arm amplification for *spxA2* gene deletion |
| 4s-down-*spxA2*-R | CTATCATGGGATAGAAAGTGAGGCATAAGTGAGAGTCAC |  |
| *spxA2*-complementary-F | CGACGGCCAGTGAATTCTCAAAGTGCTGCACGAAG | Complementation vector construction for *spxA2* gene |
| *spxA2*-complementary-R | ACGCCAAGCTTGCATGCATGATTAAGATTTATACTATTTCAAG |  |
| *liaR*-interior-F | CTTGTTGATGACCATGAG | Internal verification of Δ*liaR* mutant strain |
| *liaR*-interior-R | CAACTGCTTGAGTTCG |  |
| *liaR*-exterior-F | CGTTTGTGGCTGATTGAAC | External verification of Δ*liaR* mutant strain |
| *liaR*-exterior-R | GTTCCTCCAGGGCATAC |  |
| 4s-up-*liaR*-F | CGACGGCCAGTGAATTCGACAGCAAGTCTACAA | Upstream homology arm amplification for *liaR* gene deletion |
| 4s-up-*liaR*-R | CATTAACATAGTGCCTCCCTCACTCTGATTTAC |  |
| 4s-down-*liaR*-F | GTAAATCAGAGTGAGGGAGGCACTATGTTAATG | Downstream homology arm amplification for *spxA2* gene deletion |
| 4s-down-*liaR*- R | TGATTACGCCAAGCTTCAGCTAGTCCAGCATAAT |  |
| *liaR*-complementary-F | ACGCCAAGCTTGCATGCATGGATAAAATAAAAATTGTACTTGTTGATGAC | Complementation vector construction for *liaR* gene |
| *liaR*-complementary-R | GACGGCCAGTGAATTCTTCATCATCTTGTGGTACCAAATGG |  |
| pet28a-*liaR*-F | GAGTGCGGCCGCAAGCTTTTCATCATCTTGTGGTACC | Amplification of *liaR* CDS for construction of pET28a‑*liaR* expression plasmid |
| pet28a-*liaR*- R | TGGGTCGCGGATCCATGGATAAAATAAAAATTGTACTTGTTGATGACC |  |
| *spxA2*-*liaR*-F | GCCTTCTCACTTTCTATCCC | The region of promoter *spxA2* for EMSA |
| *spxA2*-*liaR*- R | GACAATGCTATTGAAATTGAGG |  |
| QI-F | GAGGCACTATGTTAATG | The region of negative promoter of EMSA |
| QI- R | CTTGAACTTCTTTTGG |  |
| *gyrA*-qpcr-F | CGGGACACGTACAGGCTACT | qRT-PCR analysis of *gyrA* gene expression |
| *gyrA*-qpcr-R | CGATACGAGAAGCTCCCACA |  |
| *spxA2*-qpcr-F | AAAACTTGGCTAAATGCTC | qRT-PCR analysis of *spxA2* gene expression |
| *spxA2*-qpcr-R | GAATAGAGCGAGGTAGAA |  |
| *dltC*-qpcr-F | CCTATTTATGGAGGATGT | qRT-PCR analysis of *dltC* gene expression |
| *dltC*-qpcr-R | CCAGTCATTACGACCAAA |  |
| *dltR*-qpcr-F | TATTCAGGCTTTATTGGC | qRT-PCR analysis of *dltC* gene expression |
| *dltR*-qpcr-R | CAGCTTCCCTCACTCTTT |  |
| *spxA1*-qpcr-F | CTTGGCTAAGTAAGCATG | qRT-PCR analysis of *spxA1* gene expression |
| *spxA1*-qpcr-R | AAATAATTGGGCGTCGTA |  |

**Table S3 Genes associated with bile salt hypersensitivity identified in the transposon mutant library screen**

| Mutant ID | Locus tag | Gene name | Insertion site |
| --- | --- | --- | --- |
| O23 | SAHN016_RS03270 | 606221..607426 | 606,364 |
| O77 | SAHN016_RS03285 | *ezrA* | 610,283 |
| O157 | SAHN016_RS04240 | *sodA* | 818,988 |
| L34 | SAHN016_RS09865 | *spx* | 1,973,025 |
| P230 | SAHN016_RS07300 | *dagK* | 1,466,069 |
| P199 | SAHN016_RS06725 | *aroD* | 1,339,054 |
